# Study of the probability of resistance to phage infection in a collection of clinical isolates of *P*s*eudomonas aeruginosa* in relation to the presence of Pf phages

**DOI:** 10.1128/spectrum.03010-24

**Published:** 2025-02-05

**Authors:** Lucía Blasco, Clara Ibarguren-Quiles, Carla López-Causape, Lucía Armán, Antonio Barrio-Pujante, Inés Bleriot, Olga Pacios, Laura Fernández-García, Concha Ortiz-Cartagena, Rafael Cantόn, Antonio Oliver, María Tomás

**Affiliations:** 1 Grupo de Microbiología Traslacional y Multidisciplinar (MicroTM)-Servicio de Microbiología Instituto de Investigación Biomédica A Coruña (INIBIC); Hospital A Coruña (CHUAC); Universidad de A Coruña (UDC), A Coruña, Spain; 2 Grupo de Estudio de los Mecanismos de Resistencia Antimicrobiana (GEMARA) formando parte de la Sociedad Española de Enfermedades Infecciosas y Microbiología Clínica (SEIMC), Madrid, Spain; 3 MEPRAM, Proyecto de Medicina de Precisión contra las resistencias Antimicrobianas, Madrid, Spain; 4 Servicio de Microbiología, Hospital Universitario Son Espases-IdISBa, Palma de Mallorca, Spain; 5 CIBER de Enfermedades Infecciosas (CIBERINFEC), Instituto de Salud Carlos III 38176 , Madrid, Spain; 6 Servicio de Microbiología, Hospital Universitario Ramón y Cajal and Instituto Ramón y Cajal de Investigación Sanitaria (IRYCIS), Madrid, Spain; The Ohio State University College of Dentistry, Columbus, Ohio, USA

**Keywords:** *Pseudomonas aeruginosa*, Pf phage, filamentous phage, anti-phage defense mechanisms, phage resistance

## Abstract

*Pseudomonas aeruginosa* is a bacterial pathogen that is a major cause of lung infections in cystic fibrosis (CF) and other patients. Isolates of *P. aeruginosa* from CF patients commonly carry filamentous phages (Pf phages), which constitute a family of temperate phages known to be related to biofilm production and antibiotic sequestration. In this study, we identified 12 new Pf phage genomes in a collection of clinical isolates of *P. aeruginosa* from CF patients. Study of the anti-phage defense systems in the bacterial isolates revealed the presence of 89 such systems, of which eight were encoded in the Pf phage genomes. Finally, although a weak relation between resistance to phage infection and the number of anti-phage defense systems was detected, it was observed that the phage resistance was related to the presence of Pf phages and the anti-phage defense systems encoded in these phages.

**IMPORTANCE:**

Bacteria harbor a wide range of defense mechanisms to avoid phage infections that hamper the application of phage therapy because they can lead to the rapid acquisition of phage resistance. In this study, eight anti-phage defense systems were found in the genome of 12 Pf phages that were presents in 56% of the CF isolates of *P. aeruginosa*. The high prevalence of these phages underlines the importance of our findings about newly discovered filamentous phages and the role of these phages in resistance to phage infections. Thus, the knowledge of the anti-defense system in the Pf phage genomes could be useful in assessing the possible application of phage therapy to treat an infectious disease.

## INTRODUCTION

The interaction between bacteria and the viruses that infect them, phages, is an evolutionary driving force. Both organisms coevolve in an “arms race,” with many different immune mechanisms developed in bacteria and the counterpart mechanisms developed in phages.

The filamentous phages belong to the order *Tubulavirales* (1). These phages are unique both in their morphology and life cycle. They are present in the host genome (as a specific prophage), and when assembled, they exit the cell by extrusion without lysing the bacteria, causing chronic infections (2). They have a helical structure composed of the major coat protein, which surrounds circular, positive-sense, single-stranded DNA. Many phage species integrate their genome into the host genome, but others, such as episomal phages, are non-integrative (1, 3).

The filamentous phages are widely distributed in the multiresistant pathogen *Pseudomonas aeruginosa*. This important pathogen, designated “high risk” by the World Health Organization (WHO) in 2017 (4), can cause severe infections in hospitals and is responsible for chronic infections in the respiratory tract, wounds, and burns (5). *P. aeruginosa* is closely associated with infections in cystic fibrosis (CF) patients, at least partly because of its ability to form dense biofilms, which favors the development and chronicity of the infection (5). The filamentous phages are found within the biofilms as crystal liquid structures called tactoids, which can enhance phage tolerance to antibiotics by forming an adsorptive diffusion barrier (6). The filamentous phages identified in *P. aeruginosa* are designated Pf phages, of which seven types have been described to date. The Pf phages belong to the *Inoviridae* family, which is divided in two classes, Class I and Class II, depending on X-ray diffraction of the capsid (7). It is estimated that 50%–60% of *P. aeruginosa* isolates are lysogenized by Class II Pf phages. The high prevalence of these phages in *P. aeruginosa* is related to their role in pathogenesis, virulence, and immune system evasion (2).

Pf4 is a filamentous phage that infects *P. aeruginosa* reference strain PAO1 and whose genomic structure is typical of the integrative *P. aeruginosa* filamentous phages. The genome of Pf4 and other Pf phages is divided into a conserved part, the core genome, and a non-conserved part, the accessory genome. The core genome is the part of the genome required for the completion of a replication cycle in gram-negative hosts and comprises genes related to structure, replication, assembly, and secretion (8). By contrast, the accessory genome is a variable part of the genome, which carries genes of unknown function and also toxin genes, whose functions are related to interaction with the host and involve virulence factors or toxin-antitoxin systems (8).

The anti-phage defense mechanisms, which are considered the “prokaryotic immune system,” are mainly encoded in mobile elements in the bacterial genome, such as defense islands and prophages (2). These anti-phage defense systems are frequently organized in gene clusters (9). As these systems are present in mobile genetic elements, they are usually acquired by bacteria through horizontal gene transfer, promoting environmental adaptation of the bacterial communities (10). Different anti-phage defense systems are used in immune strategies by bacteria and include the following: (i) adsorption resistance, which is the first barrier to infection. Bacteria can evade adsorption by hiding the receptors with extracellular polymers or by mutations in the receptor gene. These mutations involve the loss of receptors or structural changes in the receptors (11). (ii) Prevention of host takeover, which occurs after phage adsorption and prevents irreversible takeover of the host metabolism (11). This can be achieved by Restriction-Modification (RM) systems, which are conformed by a restriction endonuclease and a methyltransferase. RM systems act by restricting the phage genome and methylation of the host genome, thus protecting it from the endonuclease action. The Clustered Regularly Interspaced Short Palindromic Repeats (CRISPR)-associated proteins (CRISPR-Cas) form an adaptative immune system characterized by the acquisition of small fragments of foreign DNA, known as spacers, between the CRISPR locus repeats. The spacers are used to recognize exogenous nucleic acids, which are degraded by the Cas endonuclease (12). Superinfection exclusion (Sie), a defense system developed by prophages or plasmids present in the host, blocks the uptake of phage nucleic acid into the cytoplasm (10, 13). (iii) Abortive infection systems (Abi systems), which are different systems that inhibit the infection at any of the stages of DNA replication, translation, or transduction, so that phages are unable to infect the bacteria, and the bacteria die or become persistent. (iv) The Toxin-Antitoxin (TA) system, which acts by reducing bacterial metabolism and thus inhibiting phage replication under stress conditions (10–14).

The interactions between phages and bacteria are essential for the development of the phage therapy, and in this context, the knowledge of the mechanisms of resistance to phages could be used to optimize the selection of the phages that will be employed in the treatment of infections. In order to increase this knowledge in the case of CF patients, in this study, we examined the prevalence of Pf phages in 75 clinical isolates of *P. aeruginosa* from 25 chronic CF patients; due to the high prevalence of Pf phages and the presence of anti-phage resistance systems encoded in them, we also examined how the anti-phage defense systems and the presence of Pf phages are related to host resistance to phage infection.

## RESULTS

### Prevalence of Pf phages in clinical isolates of *P. aeruginosa* from CF patients

The genome of 75 clinical isolates of *P. aeruginosa* from 25 chronic CF patients (three isolates per patient) were analyzed to search for complete genomes of filamentous phages. A study of the presence of prophages in these genomes was done with PHASTEST, which reported the presence of complete genomes of 158 complete genomes of prophages, of which 42 corresponded to filamentous phages. The filamentous phages were distributed in 39 isolates, whereas 36 isolates did not carry any filamentous phage genome (Table 1). The presence of filamentous phages in all isolates derived from one patient was variable; thus, in 40% of the patients, all isolates carried a filamentous phage; in 12% of patients, the filamentous phage was present in two isolates, and in the other 12%, only one isolate carried a filamentous phage. Finally, in 36% of the patients, none of the isolates contained a filamentous phage in the genome (Table 1). When no filamentous phage was present in one isolate but was present in the other isolates of the same patient, it was observed that they belong to different sequence type (ST) (except in the case of patient 24).

**TABLE 1:** Clinical isolates of *P. aeruginosa* recovered from CF patients^*b*^

Patient	*P. aeruginosa* CF isolate	ST	Pf isolate^*c*^	N° prophages^*a*^
01	01–0440	1089	Pf01-0440	1
	01–5978	1089	ND	1
	01–7071	1089	Pf01-7071	1
02	02–5135	312	Pf02-5135a;Pf02-5135b	1
	02–5867	312	Pf02-5867a;Pf02-5867b	1
	02–6433	312	Pf02-6433a;Pf02-6433b	1
03	03–0062	285	Pf03-0062	0
	03–5302	285	Pf03-5302	0
	03–5453	285	Pf03-5453	0
04	04–5265	274	Pf04-5265	1
	04–7991	274	Pf04-7991	1
	04–8869	274	Pf04-8869	1
05	05–2269	NEW1	ND	0
	05–2840	NEW1	ND	1
	05–4672	360	Pf05-4672	2
06	06–6855	242	ND	2
	06–7209	242	ND	2
	06–9800	242	ND	2
07	07–1155	279	ND	2
	07–5966	279	ND	2
	07–8998	279	ND	2
08	08–1318	NEW2	Pf08-1318	2
	08–4371	NEW2	Pf08-4371	3
	08–5924	NEW2	Pf08-5924	3
09	09–0786	1109	Pf09-0786	2
	09–3048	1109	Pf09-3048	1
	09–9593	1109	Pf09-9593	1
10	10–6443	360	Pf10-6443	2
	10–6518	360	Pf10-6518	2
	10–7858	360	Pf10-7858	4
11	11–4349	198	ND	1
	11–7257	2475	Pf11-7257	2
	11–8664	2475	Pf11-8664	2
12	12–0969	277	ND	1
	12–2742	277	ND	1
	12–2760	277	ND	1
13	13–0154	1123	Pf13-0154	1
	13–1387	1123	Pf13-1387	1
	13–2748	1123	Pf13-2748	1
14	14–4114	319	ND	3
	14–4688	319	ND	3
	14–5818	319	ND	3
15	15–4963	412	ND	4
	15–7676	412	ND	3
	15–8860	412	ND	4
16	16–0109	252	ND	3
	16–2856	252	ND	3
	16–4264	408	Pf16-4264	6
17	17–0755	274	ND	0
	17–3115	274	ND	0
	17–8321	274	ND	0
18	18–6285	312	Pf18-6285	1
	18–7126	312	Pf18-7126	0
	18–9439	312	Pf18-9439	1
19	19–0943	1092	Pf19-0943	1
	19–6618	1092	Pf19-6618	1
	19–9746	1092	Pf19-9746	1
20	20–0447	1072	Pf20-0447	3
	20–3695	1072	Pf20-3695	3
	20–6028	1072	Pf20-6028	2
21	21–2955	198	ND	1
	21–4234	198	ND	1
	21–9889	198	ND	2
22	22–5179	NEW3	ND	1
	22–5546	2101	ND	2
	22–5835	1134	ND	0
23	23–2344	701	ND	0
	23–6966	CC701	ND	1
	23–9557	701	ND	0
24	24–0501	274	Pf24-0501	0
	24–1092	274	Pf24-1092	1
	24–7416	274	ND	1
25	25–6546	1072	Pf25-6546	3
	25–7986	235	ND	1
	25–9260	1613	ND	2
*P. aeruginosa* reference strain		Origin		
PA01		This group		
PA14		This group		
CECT 110		This group		
Lytic Phages		Origin		
φDCL-PA6		Contaminated river water (Contreras research group, UNAM) (15)		
φDCL-PA6α		Contaminated river water (Contreras research group, UNAM) (15)		
PAC8		Contaminated river water (Contreras research group, UNAM) (15)		
PAC2		Compost (This group)		

^*a*^ No filamentous phages.

^*b*^ The isolates are grouped by patient, the ST and the filamentous phage found in each isolate. The lytic phages used in the study are also shown. The clinical isolates of *P. aeruginosa* were provided by the research group led by Oliver (16)

^*c*^ ND: Not detected.

All of the Pf phage genomes were found to be integrated in an attachment site (attB) corresponding to a tRNA. Three different types of tRNA were identified as attachment sites: 45% Pf genomes were integrated in a tRNA-Met, 47.5% in a tRNA-Gly and 7.5% in a tRNA-Sec (Table 2).

**TABLE 2:** 12 Pf phages identified in this study, showing the genome size, Genbank code, ST related to each phage, insertion site for each Pf phage, CDS number, Core Genome CDS, Accessory Genome CDS and Defense System found in each Pf phage^*a*^

Pf phage	No of isolates (patients)	Isolate ST	Genbank	tRNA insertion site	Genome size	CDS	Core CDS	Accessory CDS	Defense system
**PfAC01**	7 (4)	1089/274	OR863249	tRNA-Met	11915	19	15	4	Retron; PfsE
**PfAC02a**	6 (3)	312	OR790968	tRNA-Met	13430	22	15	7	ShosTA; Kiwa; PfsE
**PfAC02b**	3 (1)	312	OR790969	tRNA-Gly	13720	18	14	4	Gabija; PfsE
**PfAC03**	3 (1)	285	OR801191	tRNA-Gly	12443	22	18	4	TA
**PfAC05**	4 (3)	360	OR801193	tRNA-Gly	12329	21	14	7	TA; PfsE
**PfAC08**	3 (1)	NEW2	OR818368	tRNA-Gly	14433	20	15	5	IetAS; PfsE
**PfAC09**	3 (1)	1109	OR818369	tRNA-Gly	10911	17	15	2	TA; PfsE
**PfAC11**	2 (1)	360	OR863245	tRNA-Met	12311	19	16	3	Avs; PfsE
**PfAC13**	3 (1)	2475	OR863246	tRNA-SeC	10211	15	14	1	**AbiEii toxin**
**PfAC16**	1 (1)	1123	OR863247	tRNA-Gly	11376	19	15	4	TA; PfsE
**PfAC19**	3 (1)	408	OR863248	tRNA-Gly	12922	20	15	5	TA; PfsE
**PfAC20**	4 (3)	1072	PP058144	tRNA-Met	13600	18	12	6	**Cytosine methyltransferase**

^*a*^ The proteins from incomplete anti-phage defense systems are highlighted in bold.

As the filamentous phages present in *P. aeruginosa* are known as Pf phages, the phages identified in this study will be named in the same way as Pf phages in general, with the number of isolates added, as appropriate (Table 1).

### Phylogenetic analysis

**Fig 1:**
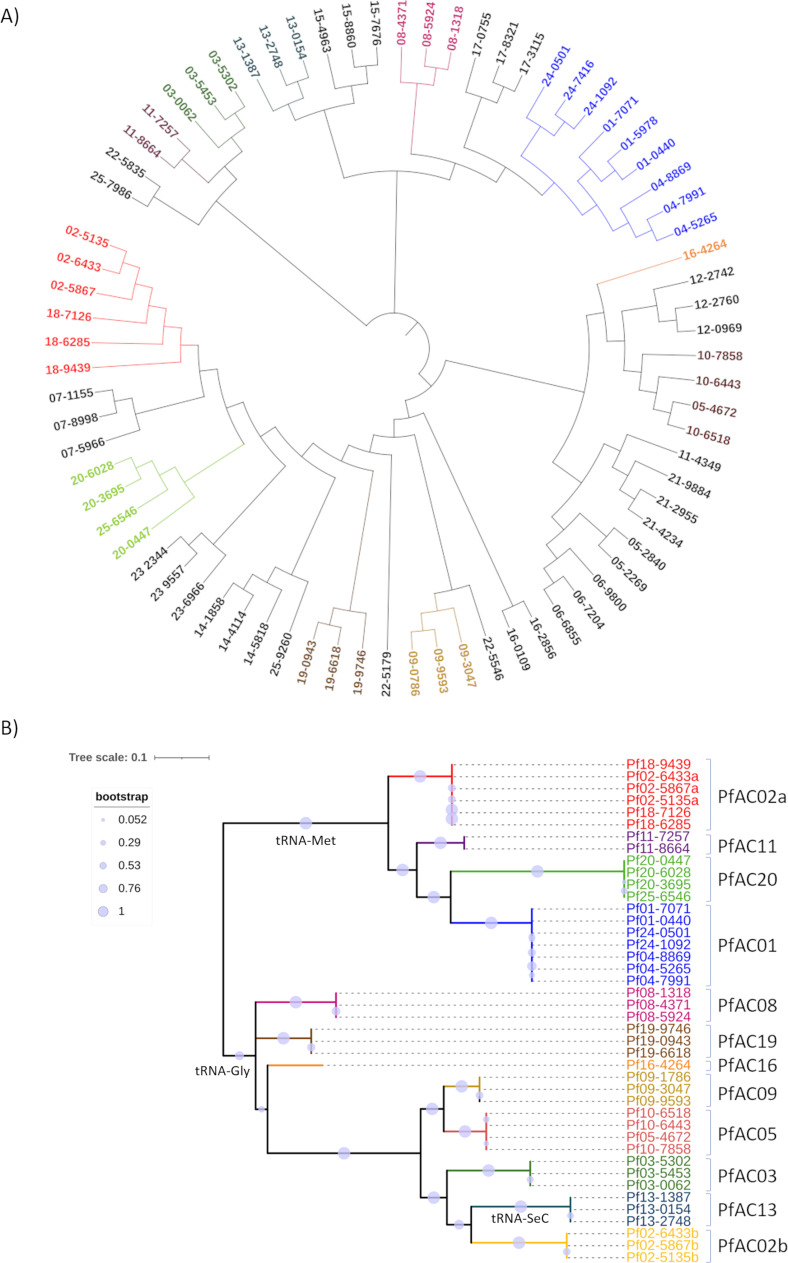
Phylogenetic analysis. (**A**) Phylogenetic tree showing clustering of the 75 clinical isolates of *P. aeruginosa*. (**B**) Phylogenetic analysis by maximum likelihood, with the tree showing the groups that corresponded to the final Pf phages identified and two major groups corresponding to the tRNA insertion sites.

The genomes of 75 clinical isolates of *P. aeruginosa* were analyzed phylogenetically, resulting in a tree with the isolates grouped by STs, which corresponded to the isolates from the same patient when they share the ST (Fig. 1A; Table 1).

**Fig 2:**
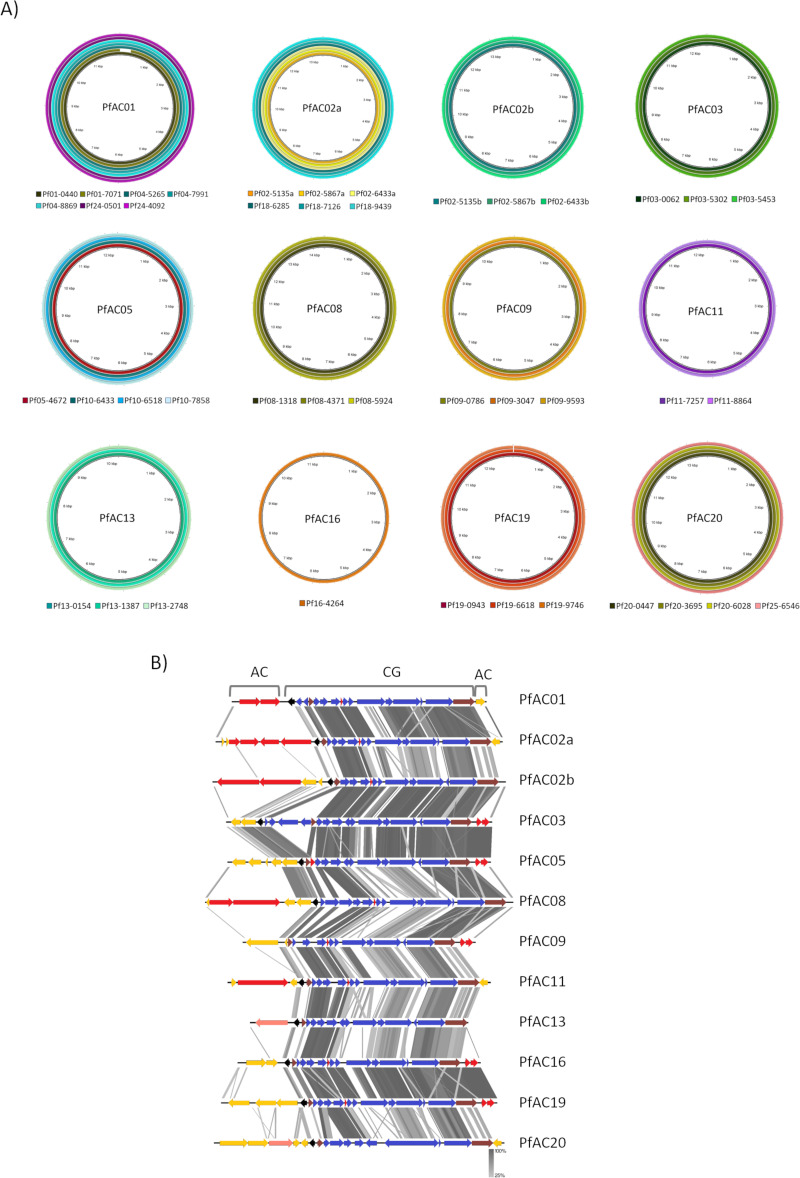
Genomic analysis of the Pf phages identified. (**A**) Brig homology analysis of the groups of filamentous phage genomes. (**B**) Protein annotation and homology of the 12 Pf phages. The core genome (CG), accessory genome (AG), integrase and excisionase (brown), excisionase negative regulator (black), anti-phage defense systems (red), hypothetical genes in the accessory genome (yellow), and genes of the core genome (blue) are shown.

The 42 Pf phage genomes identified were phylogenetically by a maximum likelihood tree. The results showed that Pf genome clustering corresponded to that observed for the phylogeny of the isolates, that is, by ST and patient (Fig. 1B and Table 2). The first cluster was constituted by the Pf genomes located in patients 01, 04, and 24. A second cluster consisted of the Pf phages identified in patients 02 and 18, and a third cluster was formed by the phages isolated from patients 20 and 25. A fourth cluster included the phages isolated from patients 10 and 05 (Fig. 1B and Fig. 2A). Brig BLAST and ANI studies comparing the phage genome sequences of each tree clade revealed a high level of homology, of between 99.92% and 100%, and the sequences were therefore assumed to be of the same phage (Fig. 2A). Based on these results, a total of 12 Pf phages were identified. The genome sequences are included in Bioproject PRJNA1082103 (Table 2).

The maximum likelihood tree was divided into two major groups corresponding to the bacterial attachment site (attB) of the prophages. One group was constituted by the Pf genomes that use the tRNA-Met as attB, and the other group included those with the tRNA-Gly and tRNA-Sec attB sites. Four of 5 phages with a tRNA-Met attB were present in isolates from different patients, whereas the phages with the tRNA-Gly and tRNA-Sec were only present in the isolates from one patient, with the exception of phage PfAC05, which was present in two patients (Fig. 1B; Table 2).

### Genomic analysis of the filamentous Pf phages

The genomic analysis revealed that all of the filamentous phage genome sizes ranged between 10 kb and 14 Kb in size and between 15 and 22 coding DNA sequences (CDS).

Gene annotation of the 12 Pf phages showed that the genome structure comprised a core genome composed of 12–18 CDS, flanked by 1–7 CDS corresponding to the accessory genome (Fig. 2B; Table 2). The organization of the core genome of the Pf phages identified was similar to that of the Pf4 phage, which is widely used as a Pf model. The following common genes were identified: C repressor gene pf4r (PA0715); excisionase XisF4 (PA0716); single-stranded DNA binding protein (PA0720); coaB, a major coat protein (PA0723); coaA, a minor coat protein (PA0724); Zot domain protein (PA0726); replication initiation protein (PA0727); and integrase intF (PA0728) (5, 17). The accessory genome flanked the core genome and was composed of moron genes, which are non-essential bacteriophage genes that benefit the bacteria. In this case, most of these genes are related to anti-phage defense, although some also encode hypothetical proteins, ATP-binding proteins, and Arc family DNA-binding proteins, which are repressors involved in the prophage lysis/lysogeny decision (18).

### Study and analysis of the anti-phage defense systems in the isolates and in Pf genomes

**Fig 3:**
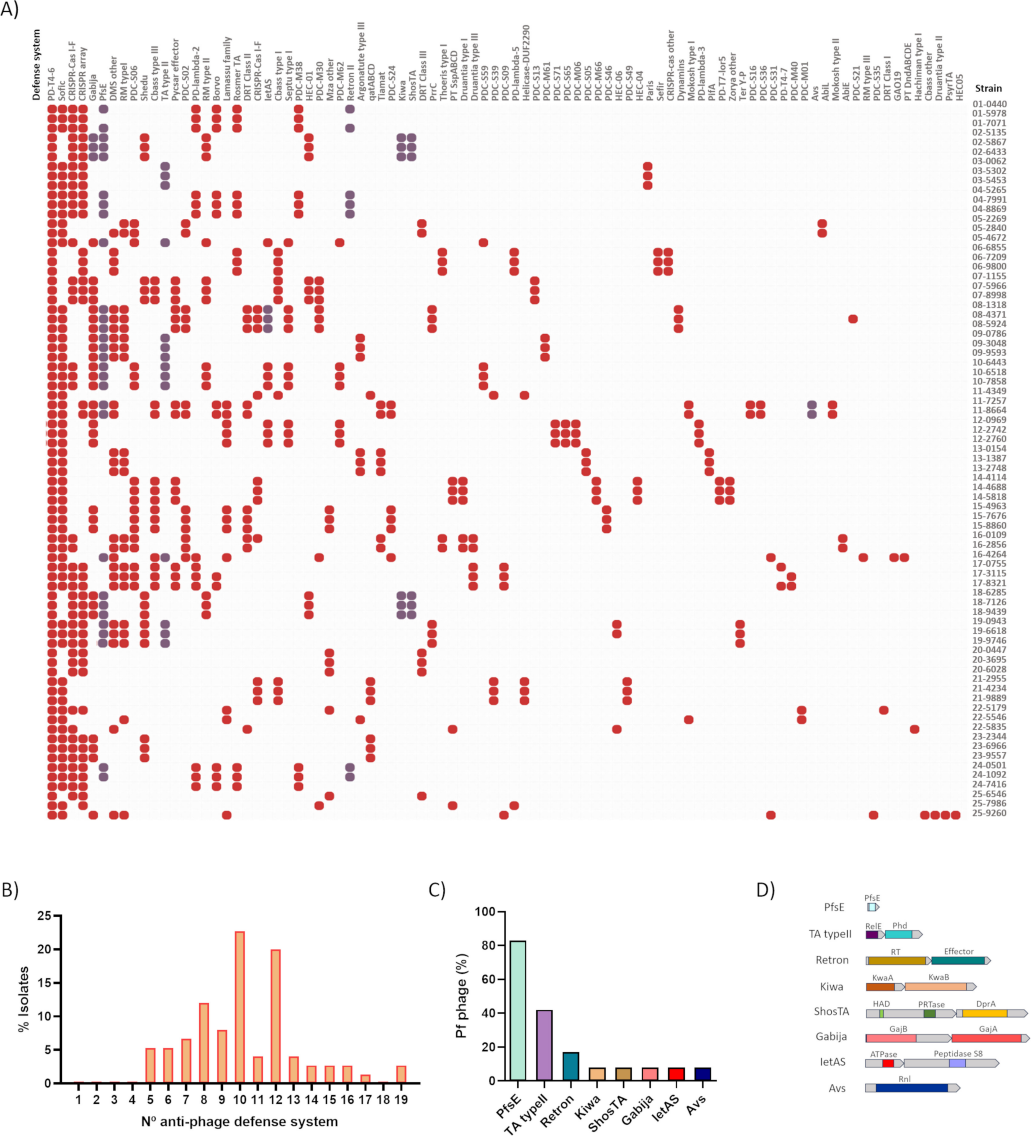
Anti-phage defense system analysis. (**A**) Distribution of the anti-phage defense systems identified by PADLOC in the genome of the 75 clinical isolates of *P. aeruginosa*. The systems ordered by prevalence in the isolates. Red spots represent those systems in the bacterial genome, and purple spots represent the systems found in the genome corresponding to a Pf phage. (**B**) Number of anti-phage defense systems per isolate. (**C**) Prevalence of each defense system in the 12 Pf phages identified. (**D**) Protein components of the anti-phage defense systems.

The genomes of 75 clinical isolates of *P. aeruginosa* were analyzed by PADLOC to search for anti-phage defense systems. A total of 89 anti-phage defense systems were detected at a frequency of between 5 and 19 per isolate (Fig. 3A and B). Isolates encoding 10 and 12 anti-phage defense systems were the most common, representing 22% and 20% of the total, respectively. Classification of the anti-phage defense systems by their strategies showed that 32.58% belonged to Abi, 8.98% involved restriction, 3.37% involved TA, 2.24% were Sie, and the strategy was not identified for 52.80% (Table S1). Of the systems with unknown strategy, those with names starting with PD or HEC were identified by the “guilt-by-embedding” approach (via PADLOC web server).

From the total of the anti-phage defense systems found in the genomes of the clinical isolates, eight were encoded in the genomes of 10 different Pf phages, and the two remaining phages carried only one protein from a clustered system. The following anti-phage defense systems were detected in the Pf phages: TA typeII, Retron, Kiwa, ShosTA, Gabija, IetSA, PfsE, and Avs (Fig. 3C and D; Table 2). Of these systems, six were encoded only in the genomes of the Pf phages. Gabija was encoded in the genomes of 28 isolates and in the genome of PfAC02b (8%), which was detected in the isolates from patient 03. IetAS was located in the genome of seven isolates and in the genome of PfAC08 (8%), present in the genome of patient 08. It was observed that those isolates with the IetAS system also contained the Gabija system in the bacterial genome. The annotation revealed that the IetAS system was composed of a peptidase S8 (IetS) and a putative ATPase (IetA). PfsE was the only gene located in the core genome. This gene was present in 10 of the 12 Pf phages (83%). The second most common system in the Pf genome was the TA Type II system, which was present in 5 Pf phages (42%). Annotation showed that this system corresponded to a cluster composed of two contiguous genes, a RelE family toxin and a Phd family antitoxin. The retron system was present in two Pf phages (17%), and according to the protein annotation, it was a cluster constituted by a retrotranscriptase and a retron effector protein. Both Kiwa and ShosTA systems were present in the PfAC02a (8%). Kiwa was a gene cluster composed by kwaA and kwaB genes, identified by PADLOC. ShosTA was also a cluster constituted by two genes, a DNA-binding protein (DprA-like) and a phosphoribosyl transferase (PRTase). Finally, Avs was present in one Pf phage (8%). Avs was constituted by an NLR ATPase identified by HHPred.

Each of the incomplete systems was represented in 8% of the Pf phages. In the case of the Pf phage PfAC13, one protein, the putative AbiEii toxin from the AbiEII toxin-antitoxin system, and a DNA cytosin methyltransferase, which was present in phage PfAC20, were detected.

### Relation between the number of Pf phages, anti-phage defense systems, and resistance to phage infection

All 75 clinical isolates were infected with four lytic *P. aeruginosa* phages to enable the study of the relationship between phage resistance and the presence of anti-phage defense systems in the accessory genome of the Pf phages.

**Fig 4:**
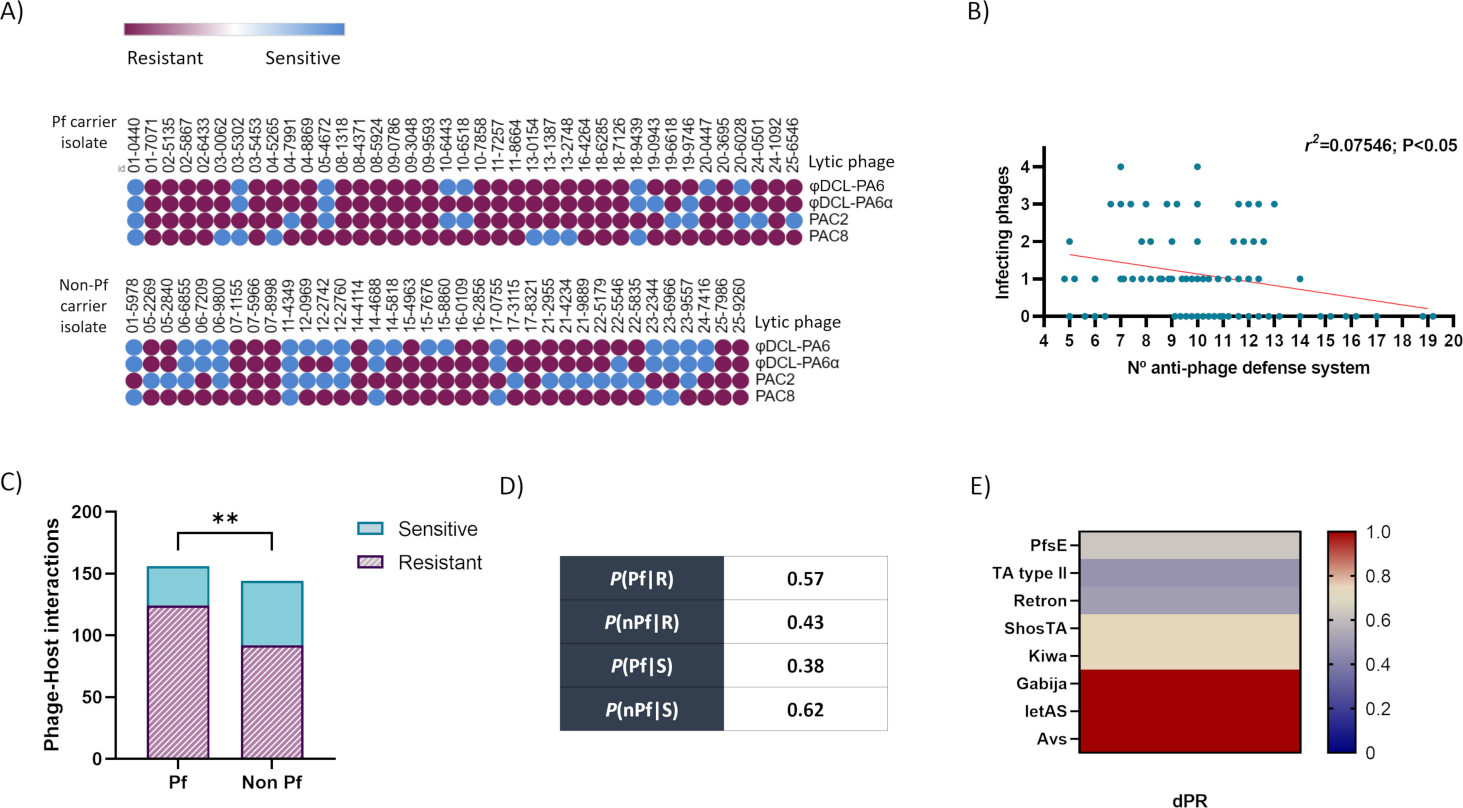
Bacterial phage resistance in relation to the anti-phage defense systems and Pf phages. (**A**) Resistance pattern for each isolate (Pf carrier and non-Pf carrier) challenged with 4 lytic phages; the graph represents the results obtained in both the spot test and infection curves. (**B**) Linear regression analysis of the correlation between the number of anti-phage defense systems and phage resistance. (**C**) Contingency analysis of the resistant and sensitive interactions between the *P. aeruginosa* identified as carriers and non-carriers of Pf phages. (**D**) Conditional probability between the presence of Pf phages in the *P. aeruginosa* isolates and the resistance or sensitivity to phage infection. (**E**) Differential probability (dPR) of each anti-phage defense system carried in the Pf genomes. Values of dPR close to 1 indicate overrepresentation of phage resistance, whereas negative values indicate overrepresentation of sensitivity to phage infection. Resistant (**R**); sensitive (**S**); Pf carrier (Pf); nPF (non-Pf carrier); defense system (DF); probability to carry a PF phage and be resistant *P*(Pf|R); probability to not carry a Pf phage and be resistant *P*(nPf|R); probability to carry a Pf phage and be resistant *P*(Pf|S); probability to not carry a Pf phage and be sensitive *P*(nPF|S); and diferential probability (dPR) of each anti-phage system.

The infection study was conducted by spot testing and broth infection curves, with those in which productive infection was observed considered positive for infection. Of a total of 300 phage-bacteria interactions, 216 (72%) were resistant, and 84 (28%) were sensitive (Fig. 4A). Linear regression analysis was conducted to relate the resistance to phage infection and the number of anti-phage defense systems in each clinical isolate, revealing a low but significant correlation between the number of anti-phage defense systems and the resistance to phage infection (*r^2^* = 0.07546 and *P* < 0.05) (Fig. 4B).

Although the number of anti-phage defense systems was weakly related to resistance, the high prevalence of Pf phages in the 75 clinical isolates of *P. aeruginosa* (56%) and the presence of anti-phage defense systems encoded in the genomes indicated the strong influence of these phages in the phage resistance pattern. The interactions between the lytic phages and 39 Pf-carrying isolates resulted in 124 resistant interactions that were significantly higher than the 99 resistant interactions with the non-Pf carrying phage. By contrast, the sensitive interactions were significantly higher in the non-Pf carriers (Fig. 4A and C). Finally, although both carrier and non-carrier Pf isolates were mainly resistant to phage infection, the calculations revealed that the presence of Pf phages increased the probability of the isolate to resist infection by a lytic phage (Fig. 4D).

The relationship between resistance and each defense system in the Pf phage carrier isolates was calculated by differential probability (Fig. 4E). All of the systems yielded dPR values > 0, suggesting that they are probably related to phage resistance (19). Gabija, IetAS, and Avs yielded the highest value of dPR (=1), which indicates that when carried in a Pf genome these systems were directly related to resistance. In the case of IetAS and Gabija, its contribution to the evasion of the phage infection was higher when located in the Pf genome than when they were encoded in the bacterial isolate genome (Fig. 3A and Fig. 4A). Also, it was observed that in relation to the isolates from patient 11, only those carrying PfAC11 (Avs) were resistant to the four lytic phages while the non-carrier isolate was sensitive to all the lytic phages. Moreover, the probability that ShosTA and Kiwa were involved in resistance was equal because these were present in the same phage, and it was not possible to differentiate the individual activity of each. By contrast, the presence of Gabija with ShosTA and Kiwa in the isolates from patient 02 increased the differential probability to 1, indicating a synergic effect. The PfsE system co-occurred with all other systems, but no synergistic effect was observed, as the differential probability was different for each phage.

### Lysogenization with Pf phages and acquisition of resistance

The lysogenization assay was done for those Pf phages carrying the anti-phage defense system with the highest dPR: PfAC02b (Gabija), PfAC08 (ietAS), and PfAC11(Avs). The three Pf phages were obtained and incubated with each of the reference strains PA01, PA14, and CECT 110. The lysogenization of the strains was tested by PCR of the anti-phage defense system of each Pf phage, finally confirming the lysogenization of PA01 strain with the phage PfAC11. No lysogens were found for PfAC02b and PfAC08 in any of the strains tested.

The low level of lysogenization can be explained by the number of resistance mechanisms present in the strains, as PA01 only have six anti-phage defense systems (1 Sofic; 1 RetronI-B; 1 PDC-S39;1 Helicase-DUF2290; 1 PD-T4-6; and 1 RM type I), whereas PA14 has 14 (1 Wadjet type I; 2 Sofic; 1 RM type II; 1 Shango; 1 PDC-S02; 1 CRISPR-Cas; 2 PDC-S06; 1 Shedu; 2 PDC-M30; 1 PD-T4-6; and 1 Gabija) and CECT110 have 11 anti-phage defense systems (3 CRISPR arrays; 1 PD-T4-6; 1 Sofic; 2 DMS other; 1 RM type I; 1 PrrC; 1 Cas I-FI; and 1 PDC-S06), all identified by PADLOC. On the other hand, the absence of specific surface phage receptors for PfA02b and PfAC08 would prevent the lysogenization of these strains.

**Fig 5:**
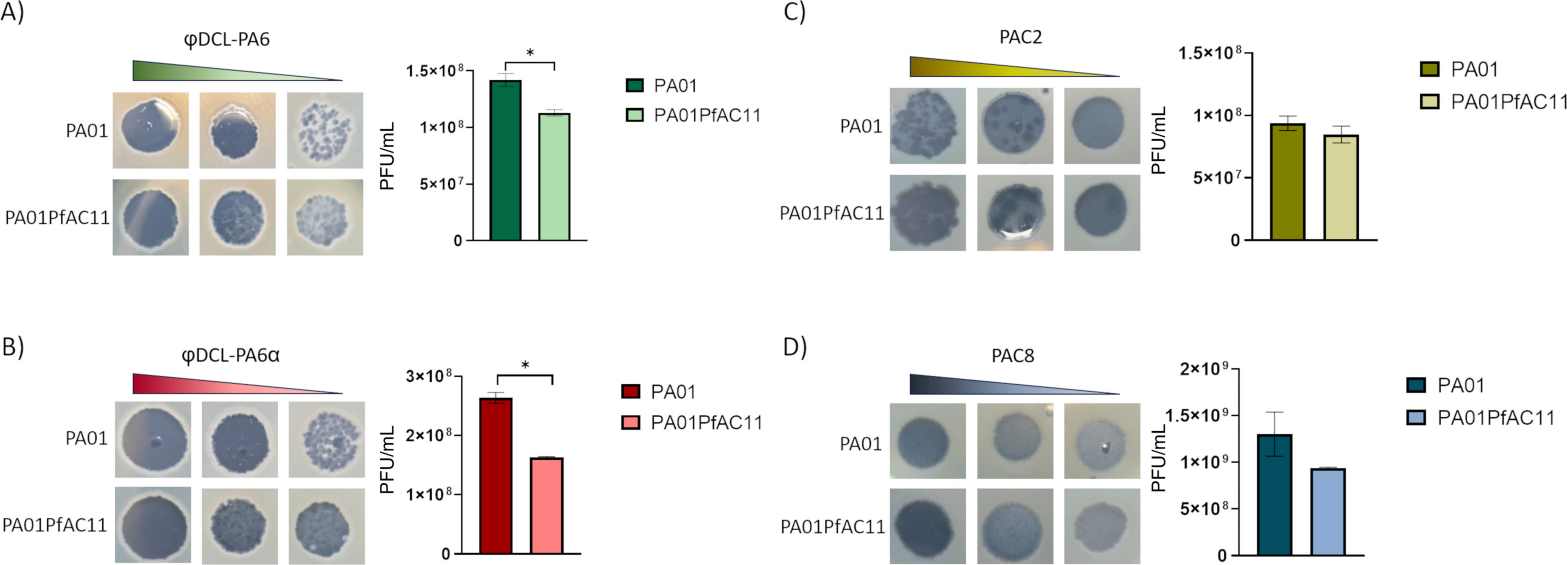
Pf phage lysogenization and acquisition of phage resistance. Spot test and PFUs counts (productive infection) for lytic phages in reference strain PA01 and PA01PfAC11 lysogen. (**A**) φDCL-PA6, (**B**) φDCL-PA6α;, (**C**) PAC2, and (**D**) PAC8.

The relation of the phage infection resistance with the lysogenization with the PfAC11 was first tested by spot test. When spots were done with serial dilutions of the four lytic phages employed in this work on a bacterial lawn of PA01 strain and PA01 lysogenized with PfAC11, the spots obtained were more turbid when done with phages φDCL-PA6 and φDCL-PA6α on the lysogenized strain (Fig. 5A and B). For the phages PAC2 and PAC8, no differences in the spots were observed (Fig. 5C and D).

The results obtained in the spot test were confirmed by testing the productive infection of the lytic phages in both PA01 and PfAC11 lysogenized PA01 strain. The acquisition of resistance by the lysogenized strain was confirmed by the counts of PFUs when it was infected by the phages φDCL-PA6 and φDCL-PA6α, as a significantly lower number of PFUs were produced in this strain when compared with those obtained in PA01 (Fig. 5A and B). As was observed in the spot test, no significant differences were obtained for phages PAC2 and PAC8 (Fig. 5C and D).

## DISCUSSION

The coexistence of bacteria and other microorganisms in the environment leads to competitive, collaborative, and predatory interactions. Bacteria have developed different systems to manage these interactions, for example, secretion systems and phage tail-like bacteriocins, which are involved in competition with other bacteria, and anti-phage defense systems, which are used to evade phage infections (10, 14, 20). The diversity of systems that make up the bacterial defense arsenal is largely driven by the “arms race” between bacteria and phages and is, in turn, enhanced by horizontal gene transfer of mobile genetic elements such as defense islands and temperate phages (21, 22). During the lysogenic phase, temperate phages maintain a symbiotic relationship with their hosts, and the fitness of both is intimately linked. The presence of virulence and defense genes in the accessory genome of the temperate phages increases the survival of both the host and the phage itself (13, 21, 23).

In this study, genomic analysis of 75 clinical isolates recovered from 25 CF patients led to the identification of 116 non-Pf prophage genomes and 42 filamentous phage genomes encompassed in the Pf-type phages. Phylogenetic relationships between the clinical isolates resulted in clustering of the isolates by ST type and by patient (when the ST was shared) (Fig. 1A). The same relationship was observed when the analysis compared the Pf phages identified in the isolates, which made it possible to group the 42 Pf phages into 12 different Pf phages, described here for first time (Fig. 1B). The genomes of these Pf phages were integrated in an attB tRNA-Met or tRNA-Gly in the same proportion and tRNA-Sec in a lower proportion, as previously reported by Fiedoruk et al. (17) (5, 17). However, we observed a relationship between the tRNA attachment site and the homology between the phages, represented in the phylogenetic tree as two major clades (Fig. 1B).

Annotation of the Pf genome and assignation of gene function revealed a canonical organization of the genes into a core genome flanked by an accessory genome, similar to that previously described for the *P. aeruginosa* Pf4, which is widely used as a Pf model. The core genome was composed of 12–18 CDS, which corresponded to the CDS identified in the core genome of the Pf4 phage and which are involved in functions of morphogenesis, assembly, DNA replication, integration, and excision. By contrast, the accessory genome was different for each phage identified, composed by 1–6 CDS, as previously described for other Pf phages (Fig. 2B and Table 2) (5, 17). The accessory genome of Pf phages, shared by other prophage families, was described as a group of genes that are not essential for the virus but with functions that benefit the host and improve its survival, such as the anti-phage defense systems or virulence genes (21). To date, only TA and PfsE anti-phage defense systems have previously been described in a filamentous phage (24). Eight anti-phage defense systems were identified in the genomes of 10 Pf phages. As previously described in other families of temperate phages, the presence of these Pf phages can enhance host survival, protecting bacteria from lytic phages via different mechanisms including inhibition of DNA translocation, premature transcription termination, and abortive infection (21). The anti-phage defense systems identified in the Pf phages in this study were involved in different defense mechanisms. From the different defense strategies, we found systems representative of Superinfection exclusion (PfsE), Abi (Retron, Kiwa, Gabija, and Avs), and TA (TA typeII, ShosTA, and IetAS) (Table S3) (2, 25).

The anti-phage defense systems identified in these Pf genomes represent 8.9% of the 89 systems identified in the collection of 75 clinical isolates of *P. aeruginosa* (Fig. 3A). The genomes of the isolates carried between 5 and 19 different anti-phage systems including those encoded in the Pf phage genomes (Fig. 3B). The variability in the number of anti-phage defense systems was found to be related to host evasion of phage infection, as higher numbers were associated with greater resistance; however, this is apparently not the only factor involved because the correlation was weak, although statistically significant (Fig. 4A and B). Similar findings were reported by Costa et al. (26), who also showed that the co-occurrence of some systems is an important factor for resistance, and by Burke et al. (27), who also observed a low but significant relation between the number of anti-phage systems and the resistance in *P. aeruginosa*. Costa et al. (26), after conducting competition assays, established that the synergy between anti-phage systems was an adaptative advantage, a consequence of an evolutive adaptation to the environmental phage pressure. Hence, bacteria can accumulate many systems with specific activity to different phages, increasing their survival in their natural environment (26). The surface phage receptors are other factors that can affect this correlation, as independently of the number of anti-phage defense systems and the presence or absence of specific receptors for the phage, will be determinants for the infection (25).

The involvement of Pf phages in bacterial phage resistance was analyzed in response to the high prevalence of these phages in the collection of *P. aeruginosa* clinical isolates. All of the Pf phages encoded more than one defense system, except PfAC03 and those with incomplete anti-phage defense systems (PfAC13 and PfAC20), with only one gene, AbiEii, of the AbiE system, and the cytosine methyltransferase form a RM system (Table 2) (28, 29). The analysis showed that although the probability of resistance was high in all the isolates, it was significantly higher in isolates carrying a Pf phage, indicating that the presence of these phages is an advantage for evasion of lytic phage infection (Fig. 4C and D). Notably, no relationship was found between the homology observed in the Pf phages and resistance to phage infection, probably because the anti-phage defense systems identified in their genome were not conserved in the phylogenetic groups.

Study of the anti-phage defense systems in the genomes of the isolates revealed the presence of 89 systems grouped into the anti-phage mechanisms of Abi, inhibition of host takeover (Sie and restriction) and also many systems with unknown mechanisms, identified as PT and PDC, which are “phage defense candidates” whose function has previously been reported as “not confirmed” (27). Except for Gabija and IetAS, the anti-phage defense systems found in the Pf genomes were only found in these phage genomes (Fig. 3A). The PfsE gene was present in 83% of the Pf phages identified and was the only gene located in the core genome, which explains its high prevalence as this region is conserved in the Pf phages (Fig. 3A through D). The PfsE protein, first identified in the Pf4 *P. aeruginosa* filamentous phage, provides resistance by Sie, suppressing the extension of the pilus type 4 (which acts as a receptor for many lytic phages) via binding to PilC (30). This protein has also been identified as an inhibitor of the quorum-sensing *Pseudomonas* quinolone signal (PQS) (31). The high prevalence of PfsE contrasts with the lower probability of involvement in phage resistance than other less frequent systems (Fig. 4E), which may be a result of its role in the suppression of the pilus as phage receptor, which would only be useful for inhibiting infection by phages using this receptor.

Of the anti-phage defense systems present in the accessory genome, the TA system was the most prevalent (43%) and was less likely to be involved in resistance than PfsE (Fig. 3A through D and Fig. 4E). Although the type II TA systems are involved in inhibiting the central cellular roles such as DNA replication and translation, they have a primarily biological role in inhibiting phage infection (6, 32). As in this study, a type II TA system was present in the Pf4 filamentous phage, in which the toxin protein belongs to the ParE family and the antitoxin to the PhD family (24, 33). As with PfsE, the high frequency of this system in the bacterial and filamentous phage genomes has favored the development of anti-TA mechanisms by lytic phages (14).

A retron system was present in 17% of the Pf phages analyzed and was estimated to have a high probability of being involved in phage resistance (Fig. 3A through D and Fig. 4E). Retrons encode a specialized reverse transcriptase and a unique chimeric single-stranded DNA/RNA molecule. Although their existence has been known for more than 30 years, it was not until 2020 that their role in phage defense was determined (34, 35). The relationship between retrons and anti-phage defense was also observed in the present study (Fig. 3D).

The other five anti-phage defense systems identified, Gabija, Kiwa, ShosTA, IetAS, and Avs, were present in 8% of the Pf phages, but their role in the resistance against phage infection was variable, with a direct relationship for Gabija, IetAS, and Avs (dPR = 1) (Fig. 3C and D and Fig. 4E). Gabija was recently partly identified as a nucleotide-sensing endonuclease (21). The Gabija system is composed of two genes, *gajA* and *gajB*, where *gajA* encodes a specific DNA-nicking endonuclease and *gajB* encodes a helicase. The GajA endonuclease is activated by the depletion of NTP and dNTP when transcription of phage DNA occurs. It has been speculated that as a helicase, GajB may interact with GajA and somehow stimulate the binding, cleavage, and/or turnover of GajA (36). The Avs system has previously been related to phage resistance and proposed to provide specific sensors for conserved structural features in phage proteins, such as the large terminase subunit and phage portal protein. It has been suggested that the Avs system tetramerizes and activates an effector-mediated Abi-like response (2). The IetAS system is also directly related to the phage resistance of the strain, but the mechanism of action remains unknown (Fig. 3C and D and Fig. 4E) (21). The low representation of these three systems in the Pf genomes contrasts with the high probability of these systems to contribute to the anti-phage resistance (Fig. 3 and Fig. 4A).

In the case of Kiwa and ShosTA, the value of the differential probability of involvement in phage defense (0.6) indicates overrepresentation of these systems in the resistant isolates (Fig. 3C and D and Fig. 4E). The ShosTA system is a TA system composed by two proteins, a DprA-like protein as an antitoxin and a phosphoribosyl transferase (PRTase) as a toxin (16). The Kiwa system was characterized as an Abi defense system constituted by two proteins, KiwaA and KiwaB. KiwaA detects inhibition of the RNA polymerases by the lytic phage proteins and activates KiwaB, which reduces the phage DNA replication in a RecBCD-dependent manner (2).

The role of the Pf phages in the anti-phage defense and phage infection resistance was confirmed for the phage PfAC11, as when a reference strain was lysogenized with this phage it acquired some resistance to the infection by the lytic phages φDCL-PA6 and φDCL-PA6α when compared with the wild-type strain, as the production of phage progeny in the lysogenized isolate was significantly lower than in the non-lysogenized isolate. Although the resistance was increased (Fig. 5A and B), this was not sufficient to completely inhibit the infection. This observation was probably due to the lower number of anti-phage defense systems in PA01 (Fig. 4B) because as was described in this and previous studies, a weak but significant relation was established between the number of anti-phage resistance systems and the probability of resistance (26, 27). The lysogenization did not confer resistance to PAC2 and PAC8, in contrast to clinical isolates (Fig. 4A; Fig. 5). This acquisition or lack of resistance, depending on the lytic phage, can be a consequence of the synergistic or antagonistic relation between pairs of anti-phage defense systems and also the anti-resistance mechanisms carried by each individual phage (25, 37). It was described by Costa et al (26) that specific anti-phage defense systems provide activity against specific phage families, which would explain the different response to the phage infection observed for the lysogenized PA01.

To our knowledge, this is the first time that all of these anti-phage defense systems encoded in Pf phages, except TA and PfsE, have been identified in this type of phage. Pf phages have been linked to virulence traits and confer a competitive and survival advantage to the bacteria, and therefore, the high prevalence of these phages in CF isolates of *P. aeruginosa* and the increased probability of these isolates being resistant to phage infection may be related to the survival and permanence of the *P. aeruginosa* isolates in the lungs of CF patients. Study of the presence of the Pf phages and the presence of anti-phage defense systems in the genomes may be of interest to improve phage therapy by facilitating the selection of appropriate lytic phages.

## MATERIALS AND METHODS

### Bacterial and lytic phage strains

Seventy-five *P. aeruginosa* clinical isolates were recovered at different times from 25 CF patients (three isolates per patient) (Table 1). The isolates, belonging to 26 STs from a collection of *P. aeruginosa* isolates from CF patients, were provided by the research group led by Oliver (Sons Espases Hospital, Palma de Mallorca, Spain) (38). *P. aeruginosa* PA01 and PA14 were used to propagate the lytic phages. Four *P. aeruginosa* lytic phages with known genomic sequences were used in the study (Table 1).

The *P. aeruginosa* isolates were cultured in LB (0.5% yeast extract; 0.5% NaCl; 1% tryptone), and agar 2% was added when necessary.

### Genome sequencing of the *P. aeruginosa* clinical isolates and filamentous phage genome identification and annotation

Next-generation sequencing (NGS) of the isolates was performed in a previous study, with the MiSeq sequencing system (Illumina platform). The sequences were assembled using the Newbler Roche assembler and Velvet (Velvet v1.2.101) (38).

The PHASTEST bioinformatic tool (39) was used to search the genomes of the isolates for phages. As this tool does not identify the phage genomes when they are located in different contigs, a manual search for those sequences at the ends of the contigs was conducted. The sequences identified by PHASTEST were also confirmed manually by searching the disrupted tRNA sites (attB). The genes were annotated using the RAST server (40), HMMER (hmmer.com), Protein BLAST (32), and HHpred (41). The anti-phage defense systems were identified using the the Procaykotic Antiviral Defense Locator (PADLOC) tool (42).

### Phylogenetic and homology study

Pangenomic and phylogenetic analyses of the genomes of the clinical isolates were conducted using the IPGA v1.09 bioinformatic tool (43).

The genome sequences of the filamentous phages were aligned using the CLUSTAL method, and a maximum likelihood tree was constructed using Molecular Evolutionary Genetics Analysis (MEGA) software, version 11 (44).

Homologous analysis of the genomic sequences was done by Average Nucelotide Identity (ANI) with the ANI calculator tool (http://enve-omics.ce.gatech.edu/ani/) and by the BLAST Ring Generator Image (BRIG) (45). Finally, the homology of the protein sequences of the phages was determined using Easyfig 2.2.5 software (46).

### Phage propagation and purification

Cultures of *P. aeruginosa* PA01 or PA14, depending on the phage propagated, were grown overnight at 37°C and 180 rpm. On the following day, the phage was propagated by the two-agar layer method (47). Briefly, the overnight culture was diluted 1:100 and incubated until the optical density at a wavelength of 600 nm (OD_600_) reached 0.5. An aliquot of 200 µL of the bacterial culture was mixed with 100 µL of the phage of interest. Soft TA (0.5% NaCl; 1% tryptone; 0.4% agar) was then added, and the mixture was spread over a TA solid agar layer. The plates were incubated at 37°C for 24 h. The propagated phage was recovered by washing the plate with SM buffer (0.1 M NaCl, 1 mM MgSO4, 0.2 M Tris–HCl, pH 7.5); 1% chloroform was then added, and the suspension was incubated for 20 min. Finally, the lysate was centrifuged, and the supernatant containing the phages was recovered and stored at 4°C.

### Phage infection assays: spot test and infection curve

**Fig 6:**
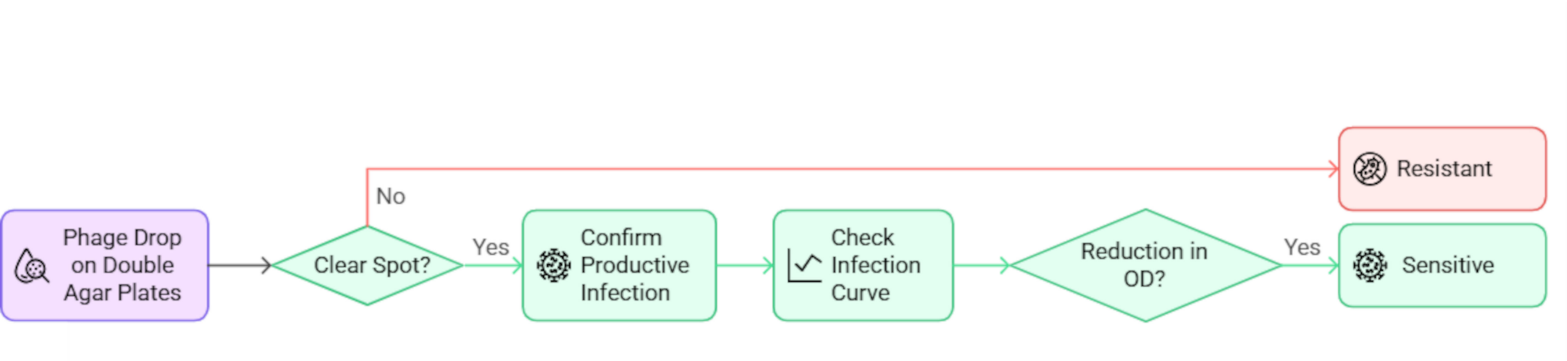
Diagram of the phage infection methodology.

A spot test was conducted, as described by Kutter et al. (48), to determine the sensitivity of the clinical isolates to the four lytic phages. Briefly, several plates were prepared by the double agar method with the host strain tested. Two microliters of a suspension containing the phage of interest (10^9^ PFU/mL) were added to the top agar. The plate was incubated at 37°C for 24 h, and the plate was examined. Infection was considered positive (sensitive) if a clear or turbid spot was observed and negative (resistant) if no spot was observed. The tests were conducted in triplicate and were considered positive when all the replicates clearly showed a spot (Fig. 6).

As some positive spots can occur as a result of abortive infection mechanisms or “lysis from without” and not from a productive infection (11), infection curve analysis in LB broth medium was conducted for the phage-host combinations that yielded positive spot tests (Fig. 6). Infection curve analysis was conducted by combining 10^7^ CFU/mL of the clinical isolate selected and 10^8^ PFU/mL of the phage selected in 200 µL of LB broth in a 96 well microplate and incubating for 24 h at 37°C in Biotek Epoch 2 (Agilent). Productive infection was assumed to have taken place when the OD was significantly lower than that of the control in the exponential phase (49).

### Statistical analysis

The correlation between the linear relationship between the number of anti-phage defense systems and phage resistance was determined, considering a 95% CI (GraphPad Prism 9.0.0).

To determine the relationship between phage resistance and the presence of Pf phages, a contingency analysis was conducted by χ^2^ test, and Fisher´s exact test was used to compute the *P* value considering a 95% CI (GraphPad Prism 9.0.0). The conditional probability of the occurrence of resistance or sensitivity to phage infection, and the presence of Pf phages was calculated as *P*(Pf|R) = *P*(PfꓵR)/*P*R, where Pf is the number of isolates carrying Pf phages, and R is the number of resistant isolates. When the same probability was calculated for non-Pf carrying isolates, nPf was used instead of Pf, and when the probability was calculated for sensitive isolates, S was used instead of R.

The relationship between the presence of complete anti-phage defense systems and the phage resistance of the host was also calculated as a differential probability for each system in those strains carrying a Pf phage, as dPR = *P*(DF|R)−P(DF|S), where DF is the anti-phage defense system. Values of dPR close to 1 indicate overrepresentation of anti-phage defense systems in resistant interactions, whereas negative values indicate overrepresentation of anti-phage defense systems in sensitive strains (49).

### Pf phages propagation and lysogenization of *P. aeruginosa* reference strains

Those Pf phages with the highest probability of being related to the resistance to phage infection were selected for the lysogenization of reference strains of *P. aeruginosa*: PA01, PA14, and CECT 110.

The propagation of the selected Pf phages was done as previously described by Gavric and Knezevic (50), with some modifications. Briefly, 1:100 dilution of an overnight culture of the strain carrying the selected Pf phages was done in 25 mL of LB and maintained for 48 h at 37°C and shaking at 180 rpm. After incubation, bacteria were removed from the medium containing phages by centrifugation at 12,000 rpm for 10 min. The centrifugation step was repeated three times. The supernatant was harvested and inoculated with 250 µL of an overnight culture of the selected strain and maintained for another 48 h at 37°C and constant agitation. The same procedures were repeated one more time, and finally, the supernatant with the Pf phages was harvested.

The Pf phages were precipitated with 10% PEG 8000 and 0.5 M NaCl and maintained overnight at 4°C. The phage suspension was then centrifuged at 14,000 rpm for 15 min, the supernatant was discarded, and the pellet suspended in SM buffer.

As the Pf phages are sensitive to chloroform and in order to differentiate from other phages that could also be produced, a portion of the suspension was treated with 1% chloroform for 30 min.

The host range of the precipitated phages was done on reference strains PA01, PA14, or CECT 110 by spot test as previously described by Kutter et al. (48). A dilution of the phage suspension treated and not treated with chloroform was done, and 5 µL of each dilution was dropped on a double agar. The infection of Pf phages was determined when the lytic plaques appear only in those spots that were not treated with chloroform.

The lytic plaques obtained were picked and suspended in SM buffer. A PCR, for the anti-phage defense genes of each phage (Table S2), was done to confirm the presence of the selected Pf phage in the suspension.

The lysogenization of the reference strains of *P. aeruginosa* with the selected Pf phage was done as described by Gavric and Knezevic (3). For this purpose, 10 mL LB were inoculated with 100 µL of an overnight culture of the type strain selected in the spot test. The culture was incubated overnight at 37°C and 180 rpm. The culture was centrifuged, and the pellet was recovered; it was washed two times, and the pellet was recovered and suspended in 1 mL LB. It was maintained overnight at 37°C and 180 rpm. The process was repeated twice, and finally, the bacteria were cultured in LB agar plates. The subcultivation was repeated three times in order to obtain stable lysogens. The confirmation of the lysogenization was done by PCR with the primers designed for the anti-phage defense genes (Table S2), and the results were visualized in an agarose gel.

The productive infection of the lytic phages in the reference strain and the lysogenized strain was done by counts of PFUs by the doble agar layer method described previously in this section. The results were statistically analyzed by t test considering a 95% CI (GraphPad Prism 9.0.0).

## Data Availability

The sequences obtain in this study were deposited in the Genbank database Table 2 (OR863249, OR790968, OR790969, OR801191, OR801193, OR818368, OR818369, OR863245, OR863246, OR863247, OR863248 and PP058144)

## SUPPLEMENTAL MATERIAL

Tables S1 and S2 spectrum.03010-24-s0001.pdf Table S1: Type of anti-phage defence systems identified in the genome of the 75 clinical isolates of *P. aeruginosa*. Table S2: List of PCR primers.

ASM does not own the copyrights to Supplemental Material that may be linked to, or accessed through, an article. The authors have granted ASM a non-exclusive, world-wide license to publish the Supplemental Material files. Please contact the corresponding author directly for reuse.

## References

[1] Knezevic P, Adriaenssens EM, Ictv Report C. 2021. ICTV virus taxonomy profile: inoviridae. J Gen Virol 102. doi:10.1099/jgv.0.001614 PMC8491893 34227934

[2] Mayo-Muñoz D, Pinilla-Redondo R, Birkholz N, Fineran PC. 2023. A host of armor: prokaryotic immune strategies against mobile genetic elements. Cell Rep 42:112672. doi:10.1016/j.celrep.2023.112672 37347666

[3] Gavric D, Knezevic P. 2022. Filamentous pseudomonas phage Pf4 in the context of therapy-inducibility, infectivity, lysogenic conversion, and potential application. Viruses 14. doi:10.3390/v14061261 PMC9228429 35746731

[4] Tacconelli E, Carrara E, Savoldi A, Harbarth S, Mendelson M, Monnet DL, Pulcini C, Kahlmeter G, Kluytmans J, Carmeli Y, Ouellette M, Outterson K, Patel J, Cavaleri M, Cox EM, Houchens CR, Grayson ML, Hansen P, Singh N, Theuretzbacher U, Magrini N, WHO Pathogens Priority List Working Group. 2018. Discovery, research, and development of new antibiotics: the WHO priority list of antibiotic-resistant bacteria and tuberculosis. Lancet Infect Dis 18:318–327. doi:10.1016/S1473-3099(17)30753-3 29276051

[5] Secor PR, Burgener EB, Kinnersley M, Jennings LK, Roman-Cruz V, Popescu M, Belleghem JD, Haddock N, Copeland C, Michaels LA, Vries CR, Chen Q, Pourtois J, Wheeler TJ, Milla CE, Bollyky PL. 2020. Pf bacteriophage and their impact on Pseudomonas virulence, mammalian immunity, and chronic infections. Front Immunol 11:244. doi:10.3389/fimmu.2020.00244 32153575 PMC7047154

[6] van Rossem M, Wilks S, Kaczmarek M, Secor PR, D’Alessandro G. 2022. Modelling of filamentous phage-induced antibiotic tolerance of P. aeruginosa. PLoS One 17:e0261482. doi:10.1371/journal.pone.0261482 35404965 PMC9000967

[7] Székely AJ, Breitbart M. 2016. Single-stranded DNA phages: from early molecular biology tools to recent revolutions in environmental microbiology. FEMS Microbiol Lett 363:fnw027. doi:10.1093/femsle/fnw027 26850442

[8] Mai-Prochnow A, Hui JGK, Kjelleberg S, Rakonjac J, McDougald D, Rice SA. 2015. Big things in small packages: the genetics of filamentous phage and effects on fitness of their host. FEMS Microbiol Rev 39:465–487. doi:10.1093/femsre/fuu007 25670735

[9] Doron S, Melamed S, Ofir G, Leavitt A, Lopatina A, Keren M, Amitai G, Sorek R. 2018. Systematic discovery of antiphage defense systems in the microbial pangenome. Science 359:eaar4120. doi:10.1126/science.aar4120 29371424 PMC6387622

[10] Ambroa A, Blasco L, López M, Pacios O, Bleriot I, Fernández-García L, González de Aledo M, Ortiz-Cartagena C, Millard A, Tomás M. 2021. Genomic analysis of molecular bacterial mechanisms of resistance to phage infection. Front Microbiol 12:784949. doi:10.3389/fmicb.2021.784949 35250902 PMC8891609

[11] Hyman P, Abedon ST. 2010. Bacteriophage host range and bacterial resistance. Adv Appl Microbiol 70:217–248. doi:10.1016/S0065-2164(10)70007-1 20359459

[12] Bleriot Ines, Blasco L, Pacios O, Fernández-García L, Ambroa A, López M, Ortiz-Cartagena C, Cuenca FF, Oteo-Iglesias J, Pascual Á, Martínez-Martínez L, Domingo-Calap P, Wood TK, Tomás M. 2022. The role of PemIK (PemK/PemI) type II TA system from Klebsiella pneumoniae clinical strains in lytic phage infection. Sci Rep 12:4488. doi:10.1038/s41598-022-08111-5 35296704 PMC8927121

[13] Bleriot Inés, Pacios O, Blasco L, Fernández-García L, López M, Ortiz-Cartagena C, Barrio-Pujante A, García-Contreras R, Pirnay J-P, Wood TK, Tomás M. 2024. Improving phage therapy by evasion of phage resistance mechanisms. JAC Antimicrob Resist 6:dlae017. doi:10.1093/jacamr/dlae017 38343627 PMC10854218

[14] Bleriot I, Blasco L, Pacios O, Fernández-García L, López M, Ortiz-Cartagena C, Barrio-Pujante A, Fernández-Cuenca F, Pascual Á, Martínez-Martínez L, Oteo-Iglesias J, Tomás M. 2023. Proteomic study of the interactions between phages and the bacterial host Klebsiella pneumoniae. Microbiol Spectr 11:e0397422. doi:10.1128/spectrum.03974-22 36877024 PMC10100988

[15] García-Cruz JC, Rebollar-Juarez X, Limones-Martinez A, Santos-Lopez CS, Toya S, Maeda T, Ceapă CD, Blasco L, Tomás M, Díaz-Velásquez CE, Vaca-Paniagua F, Díaz-Guerrero M, Cazares D, Cazares A, Hernández-Durán M, López-Jácome LE, Franco-Cendejas R, Husain FM, Khan A, Arshad M, Morales-Espinosa R, Fernández-Presas AM, Cadet F, Wood TK, García-Contreras R. 2023. Resistance against two lytic phage variants attenuates virulence and antibiotic resistance in Pseudomonas aeruginosa Front Cell Infect Microbiol 13:1280265. doi:10.3389/fcimb.2023.1280265 38298921 PMC10828002

[16] Stokar-Avihail A, Fedorenko T, Hör J, Garb J, Leavitt A, Millman A, Shulman G, Wojtania N, Melamed S, Amitai G, Sorek R. 2023. Discovery of phage determinants that confer sensitivity to bacterial immune systems. Cell 186:1863–1876. doi:10.1016/j.cell.2023.02.029 37030292

[17] Fiedoruk K, Zakrzewska M, Daniluk T, Piktel E, Chmielewska S, Bucki R. 2020. Two lineages of Pseudomonas aeruginosa filamentous phages: structural uniformity over integration preferences. Genome Biol Evol 12:1765–1781. doi:10.1093/gbe/evaa146 32658245 PMC7549136

[18] Breg JN, van Opheusden JH, Burgering MJ, Boelens R, Kaptein R. 1990. Structure of Arc repressor in solution: evidence for a family of beta-sheet DNA-binding proteins. Nature New Biol 346:586–589. doi:10.1038/346586a0 2377232

[19] Cumby N, Davidson AR, Maxwell KL. 2012. The moron comes of age. Bacteriophage 2:225–228. doi:10.4161/bact.23146 23739268 PMC3594210

[20] Blasco L, de Aledo MG, Ortiz-Cartagena C, Blériot I, Pacios O, López M, Fernández-García L, Barrio-Pujante A, Hernández-Garcia M, Cantón R, Tomás M. 2023. Study of 32 new phage tail-like bacteriocins (pyocins) from a clinical collection of Pseudomonas aeruginosa and of their potential use as typing markers and antimicrobial agents. Sci Rep 13:117. doi:10.1038/s41598-022-27341-1 36596850 PMC9810705

[21] Rousset F, Depardieu F, Miele S, Dowding J, Laval AL, Lieberman E, Garry D, Rocha EPC, Bernheim A, Bikard D. 2022. Phages and their satellites encode hotspots of antiviral systems. Cell Host Microbe 30:740–753. doi:10.1016/j.chom.2022.02.018 35316646 PMC9122126

[22] Bernheim A, Sorek R. 2020. The pan-immune system of bacteria: antiviral defence as a community resource. Nat Rev Microbiol 18:113–119. doi:10.1038/s41579-019-0278-2 31695182

[23] Bondy-Denomy J, Qian J, Westra ER, Buckling A, Guttman DS, Davidson AR, Maxwell KL. 2016. Prophages mediate defense against phage infection through diverse mechanisms. ISME J 10:2854–2866. doi:10.1038/ismej.2016.79 27258950 PMC5148200

[24] Li Y, Liu X, Tang K, Wang W, Guo Y, Wang X. 2020. Prophage encoding toxin/antitoxin system PfiT/PfiA inhibits Pf4 production in Pseudomonas aeruginosa. Microb Biotechnol 13:1132–1144. doi:10.1111/1751-7915.13570 32246813 PMC7264888

[25] Wu Y, Garushyants SK, van den Hurk A, Aparicio-Maldonado C, Kushwaha SK, King CM, Ou Y, Todeschini TC, Clokie MRJ, Millard AD, Gençay YE, Koonin EV, Nobrega FL. 2024. Bacterial defense systems exhibit synergistic anti-phage activity. Cell Host Microbe 32:557–572. doi:10.1016/j.chom.2024.01.015 38402614 PMC11009048

[26] Costa AR, van den Berg DF, Esser JQ, Muralidharan A, van den Bossche H, Bonilla BE, van der Steen BA, Haagsma AC, Fluit AC, Nobrega FL, Haas P-J, Brouns SJJ. 2024. Accumulation of defense systems in phage-resistant strains of Pseudomonas aeruginosa. Sci Adv 10:eadj0341. doi:10.1126/sciadv.adj0341 38394193 PMC10889362

[27] Burke KA, Urick CD, Mzhavia N, Nikolich MP, Filippov AA. 2024. Correlation of Pseudomonas aeruginosa phage resistance with the numbers and types of antiphage systems. Int J Mol Sci 25:1424. doi:10.3390/ijms25031424 38338703 PMC10855318

[28] Murphy J, Mahony J, Ainsworth S, Nauta A, van Sinderen D. 2013. Bacteriophage orphan DNA methyltransferases: insights from their bacterial origin, function, and occurrence. Appl Environ Microbiol 79:7547–7555. doi:10.1128/AEM.02229-13 24123737 PMC3837797

[29] Dy RL, Przybilski R, Semeijn K, Salmond GPC, Fineran PC. 2014. A widespread bacteriophage abortive infection system functions through a type IV toxin-antitoxin mechanism. Nucleic Acids Res 42:4590–4605. doi:10.1093/nar/gkt1419 24465005 PMC3985639

[30] Schmidt AK, Fitzpatrick AD, Schwartzkopf CM, Faith DR, Jennings LK, Coluccio A, Hunt DJ, Michaels LA, Hargil A, Chen Q, Bollyky PL, Dorward DW, Wachter J, Rosa PA, Maxwell KL, Secor PR. 2022. A filamentous bacteriophage protein inhibits type IV pili to prevent superinfection of Pseudomonas aeruginosa. MBio 13:e0244121. doi:10.1128/mbio.02441-21 35038902 PMC8764522

[31] Schwartzkopf CM, Taylor VL, Groleau M-C, Faith DR, Schmidt AK, Lamma TL, Brooks DM, Déziel E, Maxwell KL, Secor PR. 2024. Inhibition of PQS signaling by the Pf bacteriophage protein PfsE enhances viral replication in Pseudomonas aeruginosa. Mol Microbiol 121:116–128. doi:10.1111/mmi.15202 38038061 PMC10842821

[32] Kent WJ. 2002. BLAT--the BLAST-like alignment tool. Genome Res 12:656–664. doi:10.1101/gr.229202 11932250 PMC187518

[33] Patel PH, Maxwell KL. 2023. Prophages provide a rich source of antiphage defense systems. Curr Opin Microbiol 73:102321. doi:10.1016/j.mib.2023.102321 37121062

[34] Maxwell KL. 2020. Retrons: complementing CRISPR in phage defense. CRISPR J 3:226–227. doi:10.1089/crispr.2020.29100.kma 32833529

[35] Millman A, Bernheim A, Stokar-Avihail A, Fedorenko T, Voichek M, Leavitt A, Oppenheimer-Shaanan Y, Sorek R. 2020. Bacterial retrons function in anti-phage defense. Cell 183:1551–1561. doi:10.1016/j.cell.2020.09.065 33157039

[36] Cheng R, Huang F, Wu H, Lu X, Yan Y, Yu B, Wang X, Zhu B. 2021. A nucleotide-sensing endonuclease from the Gabija bacterial defense system. Nucleic Acids Res 49:5216–5229. doi:10.1093/nar/gkab277 33885789 PMC8136825

[37] Murtazalieva K, Mu A, Petrovskaya A, Finn RD. 2024. The growing repertoire of phage anti-defence systems. Trends Microbiol 32:1212–1228. doi:10.1016/j.tim.2024.05.005 38845267

[38] Sastre-Femenia MÀ, Fernández-Muñoz A, Gomis-Font MA, Taltavull B, López-Causapé C, Arca-Suárez J, Martínez-Martínez L, Cantón R, Larrosa N, Oteo-Iglesias J, Zamorano L, Oliver A, GEMARA-SEIMC/CIBERINFEC Pseudomonas study Group. 2023. Pseudomonas aeruginosa antibiotic susceptibility profiles, genomic epidemiology and resistance mechanisms: a nation-wide five-year time lapse analysis. Lancet Reg Health Eur 34:100736. doi:10.1016/j.lanepe.2023.100736 37753216 PMC10518487

[39] Wishart DS, Han S, Saha S, Oler E, Peters H, Grant JR, Stothard P, Gautam V. 2023. PHASTEST: faster than PHASTER, better than PHAST. Nucleic Acids Res 51:W443–W450. doi:10.1093/nar/gkad382 37194694 PMC10320120

[40] Aziz RK, Bartels D, Best AA, DeJongh M, Disz T, Edwards RA, Formsma K, Gerdes S, Glass EM, Kubal M. 2008. The RAST Server: rapid annotations using subsystems technology. BMC Genomics 9:75. doi:10.1186/1471-2164-9-75 18261238 PMC2265698

[41] Söding J, Biegert A, Lupas AN. 2005. The HHpred interactive server for protein homology detection and structure prediction. Nucleic Acids Res 33:W244–8. doi:10.1093/nar/gki408 15980461 PMC1160169

[42] Payne LJ, Meaden S, Mestre MR, Palmer C, Toro N, Fineran PC, Jackson SA. 2022. PADLOC: a web server for the identification of antiviral defence systems in microbial genomes. Nucleic Acids Res 50:W541–W550. doi:10.1093/nar/gkac400 35639517 PMC9252829

[43] Liu D, Zhang Y, Fan G, Sun D, Zhang X, Yu Z, Wang J, Wu L, Shi W, Ma J. 2022. IPGA: A handy integrated prokaryotes genome and pan-genome analysis web service. Imeta 1:e55. doi:10.1002/imt2.55 38867900 PMC10989949

[44] Tamura K, Stecher G, Kumar S. 2021. MEGA11: molecular evolutionary genetics analysis version 11. Mol Biol Evol 38:3022–3027. doi:10.1093/molbev/msab120 33892491 PMC8233496

[45] Alikhan NF, Petty NK, Ben Zakour NL, Beatson SA. 2011. BLAST ring image generator (BRIG): simple prokaryote genome comparisons. BMC Genomics 12:402. doi:10.1186/1471-2164-12-402 21824423 PMC3163573

[46] Sullivan MJ, Petty NK, Beatson SA. 2011. Easyfig: a genome comparison visualizer. Bioinformatics 27:1009–1010. doi:10.1093/bioinformatics/btr039 21278367 PMC3065679

[47] Kropinski AM, Mazzocco A, Waddell TE, Lingohr E, Johnson RP. 2009. Enumeration of bacteriophages by double agar overlay plaque assay. Methods Mol Biol 501:69–76. doi:10.1007/978-1-60327-164-6_7 19066811

[48] Kutter E. 2009. Phage host range and efficiency of plating. Methods Mol Biol 501:141–149. doi:10.1007/978-1-60327-164-6_14 19066818

[49] Beamud B, García-González N, Gómez-Ortega M, González-Candelas F, Domingo-Calap P, Sanjuan R. 2023. Genetic determinants of host tropism in Klebsiella phages. Cell Rep 42:112048. doi:10.1016/j.celrep.2023.112048 36753420 PMC9989827

[50] Gavric D, Knezevic P. 2021. Optimized method for Pseudomonas aeruginosa integrative filamentous bacteriophage propagation. Front Microbiol 12:707815. doi:10.3389/fmicb.2021.707815 35095778 PMC8790315

