# Supplementary material for: Study of the probability of resistance to phage infection in a collection of clinical isolates of Pseudomonas aeruginosa in relation to the presence of Pf phages
Source: Microbiol Spectr. 2025 Feb 5;13(3):e03010-24. doi: 10.1128/spectrum.03010-24 (PMC11878078; doi:10.1128/spectrum.03010-24)
Supplement: Tables S1 and S2 — Table S1: Type of anti-phage defence systems identified in the genome of the 75 clinical isolates of P. aeruginosa. Table S2: List of PCR primers. [file spectrum.03010-24-s0001.pdf]

Table S1. Type of anti-phage defence systems identified in the genome of the 75 clinical isolates of *P. aeruginosa*. The table shows the name of each phage, the percentage of each system in the isolates and the bacterial phage defence mechanism of each system. Abortive infection (Abi), Restriction, Toxin-antitoxin (TA), Superinfection exclusion (Sie) and Unknown mechanism (UK) [7,9,19,28].

Anti-phage defense system	% isolates	Anti-phage defense mechanism
**PD-T4-6**	100.00	Abi
**SoFic**	78.67	Abi
**CRISPR-Cas I-F1**	56.00	Restriction
**CRISPR array**	54.67	Restriction
**Gabija**	41.33	Abi
**PfsE**	37.33	Sie
**DMS other**	34.67	UK
**RM type I**	28.00	Restriction
**PDC-S06**	22.67	UK
**Shedu**	20.00	Sie
**Cbass type III**	20.00	Abi
**TA**	18.67	TA
**Pycsar effector**	18.67	Abi
**PDC-S02**	17.33	UK
**PD-lambda-2**	17.33	UK
**RM type II**	17.33	Restriction
**Borvo**	17.33	Abi
**Lamassu family**	16.00	Abi
**Rosmer TA**	16.00	TA
**DRT Class II**	14.67	UK
**CRISPR Cas type I-E**	14.67	Restriction
**IetAS**	13.33	Abi

**Cbass Type I**	13.33	Abi
**Septu type I**	13.33	Abi(putative)
**PDC-M38**	12.00	UK
**HEC-01**	12.00	UK
**PDC-M30**	10.67	UK
**mza other**	10.67	UK
**PDC-M62**	9.33	UK
**Retron II**	9.33	Abi
**Argonaute type III**	9.33	Abi
**qatABCD**	9.33	UK
**Tiamat**	9.33	UK
**PDC-S24**	8.00	UK
**Kiwa**	8.00	Abi
**ShosTA**	8.00	TA
**DRT class III**	8.00	Uk
**PrrC**	8.00	Abi
**Thoeris type I**	6.67	Abi
**PT SspABCD**	6.67	UK
**Druantia type I**	6.67	UK
**Druantia type III**	6.67	UK
**PDC-S59**	5.33	UK
**PDC-S39**	5.33	UK
**PDC-S09**	5.33	UK
**PD-Lambda-5**	5.33	Abi(putative)
**Helicase-DUF2290**	5.33	UK
**PDC-S13**	4.00	UK
**PDC-M61**	4.00	UK
**PDC-S71**	4.00	UK

**PDC-S65**	4.00	UK
**PDC-M06**	4.00	UK
**PDC-S05**	4.00	UK
**PDC-M66**	4.00	UK
**PDC-S46**	4.00	UK
**HEC06**	4.00	UK
**PDC-S49**	4.00	UK
**HEC-04**	4.00	UK
**Paris**	4.00	Abi
**Sefir**	4.00	Abi
**CRISPR Cas other**	4.00	Restriction
**Dynamins**	4.00	UK
**Mokosh type I**	4.00	Abi
**PD-Lambda-3**	4.00	Abi
**PifA**	4.00	Abi
**PD-T7-lor5**	4.00	Abi(putative)
**Zorya other**	4.00	UK
**Ter Y-P**	4.00	UK
**PDC-S16**	2.67	UK
**PDC-S36**	2.67	UK
**PDC-S31**	2.67	UK
**PD-T4-7**	2.67	UK
**PDC-M40**	2.67	UK
**PDC-M01**	2.67	UK
**Avs**	2.67	Abi
**AbiL**	2.67	Abi
**Mokosh type II**	2.67	Abi
**AbiE**	2.67	Abi

**PDC-S21**	1.33	UK
**RM type III**	1.33	Restriction
**PDC-S35**	1.33	UK
**DRT class I**	1.33	UK
**GAO19**	1.33	UK
**PT DndABCDE**	1.33	Restriction
**Hachiman type I**	1.33	Abi
**Cbass other**	1.33	Abi
**Druantia type II**	1.33	UK
**PsyrTA**	1.33	Abi(putative)
**HEC-05**	1.33	UK

Table S2. List of PCR primers.

Primers		
Pf phage	Gene name	Sequence (5´-3´)
PfAC02b	GajB (Forward)	ATGACTCCGTACGACGAAATTCAAG
	GajB (Reverese)	TCTCCTGCGACACATCAAAGAAC
PfAC08	Iet (Forward)	TGCAAATGGTCATGAGCTGATAGG
	ietS (Reverse)	TACGAGGGCATATGGCATGG
PfAC11	Avs (Forward)	AGTGAGGTAGCTCTTCGTGGTTTTG
	Avs (Reverse)	CCACCCCTTCTCTTTCTCACTACC
